# Amœbic Dysentery

**Published:** 1892-04-30

**Authors:** 


### The Practitioner's Mirror and Retrospect.

[*The Editor will be glad to receive offers of co-operation and contributions from members of the profession. All letters should be addressed to* The Editor, The Lodge, Porchester Square, London, W.]

### MEDICINE.

### AMŒBIC DYSENTERY.

Dysentery is a disease of which the symptoms are fairly definite, and the course often grave. In other than tropical climates, however, the term is used somewhat loosely, and often applied to many severe forms of diarrhœa other than true dysentery. Recent investigations of Drs. W. T. Councilman and A. A. Lafleur* seem to demonstrate that a group of symptoms are closely associated with the presence of amœbiform organisms, and constituting amœbic dysentery.

Summarising this disease, the authors find that it is characterised by definite anatomical lesions, having a certain uniform character, although differing in some respects in the various organs; the differences being capable of explanation by the differences in anatomical structure of the various organs, the greater number of amœbæ, and the varying intensity of their action, the complication of the action of the amœbæ by the action of other organisms, and the varying degrees of resistance of the tissue. They find no analogy between the tissue changes produced direct by the amœbæ and those produced by the bacteria. If, in general, we assume the action of the bacteria to be an injury to the tissue, shown either by a slow necrosis with the production of granulation tissue, or a more rapid necrosis with more acute inflammatory changes and the production of an exudation with fibrin and pus, the action of the amœbæ is different from either. They regard the action of the amœbæ as both a production of necrosis of the tissue and a solvent action on the intercellular substances and the connective tissue as well as the cells of the tissue.

The amœbæ, probably reach the large intestine in food or drink. They exert no action on the stomach or small intestines, for they probably do not find there the suitable conditions for their increase. It is only when they reach the large intestines that they find the conditions favourable; and one of the essential conditions seems to be, from the researches of Cunningham, and also in part from the author's, that the re-action of the material in which they grow shall be alkaline. The small intestine seems only to be affected in those cases where there is an enormous number of amœbæ in the large, and they probably enter the small from the large, but they only affect a small section of the ileum. We must believe that the amœbæ multiply rapidly in the large intestine, and to a much larger extent here than in the tissues. It is especially in the loose connective tissue between the coats of the intestine, that the action of the amœbæ is most pronounced. The muscular coat offers some resistance; it is not generally destroyed, but the amœbæ pass through it in certain places, enter the intermuscular tissue, and there play the same part as they did in the submucous tissue, the tissue overlying the infiltration undergoing necrosis in mass. Along with this there is a peculiar hyaline degeneration of the muscular coat. Not only do the ulcers increase by this continual undermining process, but there is probably a continuous softening and liquefaction of the tissue from the contents of the intestine. The whole typical course and appearance of the ulcer may be completely changed from action of the bacteria in the intestinal canal. Extensive suppuration may take place, and this process may be entirely substituted for the action of the amœbæ.

After an extensive review of the literature of dysentery, the authors conclude that no single form of dysentery is peculiar to the tropics, and entitled to be regarded as tropical dysentery. One thing seems certain—that the lesions in most of the epidemics of dysentery are those of diphtheritic colitis. Diphtheritic colitis is a distinct affection, and the lesions produced by the amœbæ have nothing to do with this, although the two forms may co exist.

As the result of the work which has been done on the subject, the authors feel justified in concluding as follows:—

A.—Amœbic dysentery is a form of dysentery which ætiologically, clinically, and anatomically should be regarded as a distinct disease.

B.—They would consider that (*a*) the amœba dysenteriæ has been shown to be the causative agent, from its constant presence in the stools and in the anatomical lesions, and from the inoculation experiments of Kartulis. (*b*) Clinically the disease is characterised by the presence of amœbæ in the stools, which, in addition, present physical characters different from those seen in the stools of other forms of dysentery, as noted above; by a variable onset, course and duration of which the special features are periods of intermission alternating with exacerbations; and by a marked tendency to chronicity, with the production of a greater or less degree of anæmia. (*c*) Anatomically, the disease is characterised by the production of ulcers in the colon, which generally differ from those found in other forms of dysentery. The ulceration is produced in infiltration of the submucous tissue and necrosis of the overlying mucous membrane, the ulcer in consequence having the undermined form. Frequently, in addition to the ulcers, there is infiltration of the submucous tissue without ulceration. In all of these lesions, unless complicated by the action of bacteria, there is absence of the products of purulent inflammation.

C.—Abscess of the liver, with or without involvement of the lung, is a frequent complication, much more so than in any other form of dysentery. The involvement of the lung may early follow hepatic involvement, and be detected by the occurrence of amœbæ in the sputum before there is evidence of liver abscess. These abscesses differ in their anatomical features from those produced by other causes. The chief difference is found in the absence of purulent inflammation, the abscess being caused by necrosis, softening and liquefaction of the tissue. In these abscesses the amœbæ are not associated with any other organisms.

D.—The disease is widely distributed, and is found in most countries in Europe, in most parts of the United States, and in the tropics everywhere.

E.—This is the form of dysentery which has been commonly called tropical dysentery.

It is impossible to do justice to the work of Dr. Councilman and Dr. Lafleur in any brief reference to their report. It will, however, be observed that their researches seem to throw light on many obscure points in the course of dysenteric cases, and their report merits careful consideration.

* Johns Hopkins Hosp. Rep., Vol. II. Path.

